# Multidisciplinary Perspectives on a New Hospital Addiction Consult Service: A Mixed- Methods Study

**DOI:** 10.21203/rs.3.rs-8149080/v1

**Published:** 2025-12-11

**Authors:** M Holliday Davis, Jessica Tolbert, Sarah Nessen, Amanda Perez, Korey Henderson, Bridget Durkin, Judy Chertok, Jeanmarie Perrone, Rachel French, Rachel McFadden, Ashish Thakrar, Samantha Huo, J Deanna Wilson, Shoshana Aronowitz, Margaret Lowenstein

**Affiliations:** University of Pennsylvania; University of Pennsylvania; University of Pennsylvania; Hospital of the University of Pennsylvania; University of Pennsylvania; Hospital of the University of Pennsylvania; University of Pennsylvania; University of Pennsylvania; University of Pennsylvania School of Nursing; University of Pennsylvania Center for Addiction Medicine and Policy; University of Pennsylvania; University of Pennsylvania; University of Pennsylvania; University of Pennsylvania School of Nursing; University of Pennsylvania

## Abstract

**Background:**

Hospital-based Addiction Consult Services (ACS) are increasingly implemented to improve care for patients with substance use disorders (SUD). While ACS are generally well-regarded, clinicians of various roles may hold different perceptions of their impact.

**Methods:**

We conducted a web-based survey of physicians, advanced practice providers (APP), and nurses at a Philadelphia academic hospital from August-September 2024, approximately 18 months years after ACS implementation. The survey assessed attitudes and perceptions of the ACS with 6 questions using a 5-point Likert scale. These data were integrated with results from semi-structured interviews with physicians, APP, and nurses from November 2023-January 2024, 7–9 months after ACS implementation, to provide a richer understanding of provider perspectives on the impact of the ACS. We used descriptive statistics to characterize the samples and analyzed survey data by clinician group chi-squared tests. Interviews were analyzed using thematic content analysis.

**Results:**

Of 793 clinicians surveyed, 311 responded (39%), including 128 nurses (41%), 108 resident physicians (35%), 49 attending physicians (16%), and 26 APPs (8%). Most providers reported that the ACS positively impacted patient care. Surveyed nurses reported significantly smaller perceived improvements in quality of care and communication compared to other clinicians (43–63% nurses vs. 77–98% other clinicians, p < 0.001, for 5 of 6 questions). While qualitative feedback was positive across groups, nursing interview narratives emphasized communication gaps, limited integration between nursing and the ACS, and a desire for additional training and education around SUD care.

**Conclusions:**

While inpatient ACS improve key aspects of SUD care across professional groups, their impact may be enhanced through intentional integration of all frontline providers. Embedding nurses or nurse educators into ACS structures, strengthening multidisciplinary collaboration, and providing standardized SUD training may enhance team cohesion and ensure that all providers feel equipped and supported in caring for patients with SUD.

## 1. INTRODUCTION

Morbidity and mortality related to substance use have increased in the United States in recent decades.^1^ Although overall overdose rates have declined from their peak in 2022, they remain disproportionately high among certain groups, particularly Black and American Indian/Native Alaskan communities.^2^ Access to treatment, including medications for opioid use disorder (MOUD), and harm reduction services, reduces drug-related morbidity and mortality.^3^ People with substance use disorders (SUD) often present to acute care settings like emergency departments and hospitals, creating a ‘reachable moment’ to connect them to treatment and other services.^4–7^

Addiction consult services (ACS) are multidisciplinary teams that provide specialized health care and social support for patients with SUD. ACS models vary but are often led by an addiction medicine or addiction psychiatry physician or advanced practice provider (APP) and may include social workers, nurses, counselors, clinical trainees, and peer specialists or others with lived experience.^8,9^ Although the ACS literature frequently highlights ACS as ‘interdisciplinary’ teams, closer review reveals that nursing roles are often absent from published ACS models. For example, a recent scoping review identified nurses in only eight of the more than 41 ACS described, notably registered nurses in only five.^10^

Implementation of ACS programs has grown nationally, and evidence supporting their effectiveness continues to build.^8,11^ Randomized control trials and other rigorous studies have demonstrated that ACS implementation can improve patient outcomes, including increased initiation and continuation of MOUD, sustained engagement in care, and reduced mortality.^7,12,13^ A systematic review by Bahji et al. found that ACS programs “create a therapeutic microcosm for highly marginalized patients.”^8^ Other studies show that multidisciplinary teams in ACS models play a critical role in supporting patients through a combination of medical and psychosocial interventions.^9,14^ Beyond their impact on patients, ACS are generally well-regarded by hospital clinicians. Prior work has shown that ACS positively influence attitudes and practices of generalist hospital providers toward patients with SUDs, including higher rates of MOUD initiation and continuation during hospitalization, improved comfort among non-addiction specialists in delivering SUD care, and increased belief in the efficacy of SUD treatment for hospitalized patients.^10,15,16^

Though there is evidence that ACS models support medical teams in delivering care, less is known about how these services meet the needs of different multidisciplinary groups within the hospital, or how and why perceptions of ACS differ across clinician roles. A recent scoping review found that physicians tend to report more positive experiences with ACS than nurses and other health providers such as social workers, who described more mixed interactions.^15^ Consult models are often oriented around physician workflows, and it is less clear how ACS can best support other staff, particularly nurses who regularly provide addiction-specific bedside care (e.g., wound care) and are often the first to respond to patient care challenges.^17^ To fill this gap, our study aim was to explore perspectives about ACS implementation among multi-disciplinary hospital-based clinicians, including physicians, APP, nurses, peer recovery specialists, and social workers. We used a mixed methods approach to evaluate clinician perspectives on the impact of a newly implemented ACS, with a focus on contextualizing variability across professional roles to identify opportunities for improved care delivery.

## 2. METHODS

We conducted a mixed methods study combining data from a clinician survey and qualitative interviews using a sequential explanatory approach in which qualitative data were used to provide an in-depth exploration of quantitative results.^18^ First, we analyzed data from a web-based multi-disciplinary clinician survey fielded one year after the implementation of an ACS at an urban, academic hospital assessing attitudes towards ACS implementation. Quantitative data were integrated with data from semi-structured interviews with clinicians from the same hospital to provide a richer perspective and contextualize survey findings. The study was approved by the University of Pennsylvania Institutional Review Board with a waiver of informed consent for the survey.

### 2.1 Study setting

The study was conducted at an urban academic hospital in Philadelphia, Pennsylvania, that implemented an inpatient ACS in April 2023. The ACS includes addiction medicine physicians, a social worker, a peer recovery specialist, and clinical trainees. The consult service supports general inpatient teams in providing treatment and discharge planning for patients with opioid and other substance use disorders, including initiation and continuation of MOUD, harm reduction education, and linkage to outpatient care. ACS team members also led the development and adaptation of health system pathways for MOUD initiation, withdrawal and pain management in patients with SUDs across hospitals.

### 2.2 Survey design and administration

The survey assessed clinician perceptions of the ACS overall and in relation to specific domains, including its impact on clinical care, harm reduction and discharge planning. Responses were collected using a 5-point Likert scale ranging from “strongly disagree” to “strongly agree.” We also collected demographic data, including professional role, years of experience, and optional self-disclosure of having family or friend with SUD. We invited all attending physicians, resident physicians, APPs, and nurses from medical and surgical floors at the study hospital to participate in the survey via email, with up to three reminders. The survey was self-administered by respondents using the REDCap secure web platform, from August to September 2024, 1 year and 4–5 months after the implementation of the ACS. Participants received a $10 incentive for completing the survey.

### 2.3 Quantitative data analysis

Survey data were analyzed using descriptive statistics to characterize overall responses and differences by clinical role. Likert-scale responses were dichotomized for analysis (e.g., combining “agree” and “strongly” into a single “agree” category). We used chi-squared tests to assess differences in proportions across provider groups. Analyses were conducted using Stata 18 (StataCorp, College Station, TX).

### 2.4 Semi-structured Interview Design and Data Collection

We developed an interview guide to explore clinician experiences working with the ACS, perceived impact of the service, and perceived barriers and facilitators to care for patients with SUD based on prior literature and preliminary work from our team.^19–21^ We recruited a purposive sample of inpatient clinicians across disciplines, including physicians, APP, nurses, peer recovery specialists, and social workers to participate in semi-structured interviews. Recruitment occurred from November 2023 to January 2024, 7–9 months after ACS implementation. Participants were invited via email by a member of the study team. Participants provided verbal informed consent and received a $50 incentive for their time. Semi-structured interviews were conducted via videoconference by members of the research team trained in qualitative interviewing. Interviews lasted 30–60 minutes, were audio-recorded and professionally transcribed.

### 2.3 Qualitative data analysis

Two members of the study team (AP, SN) analyzed the findings using thematic content analysis using NVivo 11 using a constant comparative approach to identify patterns and themes across transcripts. We did not use a predetermined theoretical framework; instead, themes were derived inductively from the data. To enhance trustworthiness and credibility of the data analysis process, kappa estimates were iteratively generated for inter-rater reliability. Seventeen percent of transcripts were double coded with a kappa of 0.8. Coding discrepancies were discussed among coders and resolved through consensus, with input from the broader study team when needed. We then used thematic content analysis to identify final themes using iterative review and consensus across the study team. In addition to identifying themes consistent across all provider groups, we examined nursing-specific perspectives to contextualize discrepancies observed in the survey data and to deepen understanding of patterns suggested by the quantitative results. The research team included multidisciplinary providers and researchers, harm reductionists, and students, whose professional and experiential backgrounds informed interpretation of the findings. This manuscript adheres to the Equator network guideline SRQR (“Standards for reporting qualitative research”).^22^

## 3. RESULTS

### 3.1 Survey respondent characteristics

There were 311 survey respondents out of a sample of 793 (response rate 39%). This sample includes 128 nurses (41%), 108 resident physicians (35%), 49 attending physicians (16%), and 26 advanced practice providers (APP) (8%) (Table 1). Respondents were majority female (73%) and predominantly identified as White (56%), followed by Asian (24%), Black (11%), and Other (9%). 71% of participants were Non-Hispanic/Latine. 28% reported having a close friend or family member with a SUD, with the highest prevalence among nurses (42%), followed by APP (35%), resident physicians (19%), and attending physicians (10%).

### 3.2 Survey Results

Across all respondents, a majority agreed or strongly agreed that the ACS positively impacted several aspects of care. For example, 80% felt the ACS positively impacted care for people who continue to use drugs, 77% felt it improved their ability to provide clinical care, and 79% felt it supported discharge planning (Table 2). However, there were statistically significant differences by professional role across these domains, with nurses consistently reporting lower levels of agreement compared to other groups (43–63% nurses vs. 77–98% other clinicians, p < 0.001, for 5 of 6 questions). While agreement was high among attendings, APP, and residents, nurses were significantly less likely to endorse these benefits of the ACS, particularly in areas such as communication (43% of nurses versus 88–96% of other groups) and perceived support for clinical care (54% of nurses versus 89–98% of others).

### 3.3 Qualitative Results

We supplemented quantitative data with qualitative data from interviews with 20 multi-disciplinary inpatient providers at the study hospital. Participants included attending physicians (n = 4), resident physicians (n = 4), nurses (n = 7), APPs (n = 2), social workers (n = 2), and a peer recovery specialist (n = 1) (Table 3). Interview participants primarily identified as White (65%), non-Hispanic or Latine (100%) and female (60%) with 1–5 years of experience (60%).

In our analysis, we found that providers across all professional groups agreed that the ACS positively impacted three features of patient care: 1) the management of pain and withdrawal in patients with SUD, 2) discharge planning and care coordination, and 3) provider moral distress and burnout associated with addiction care. However, nurse participants identified unique challenges with the ACS related to communication, role clarity, and team integration that their colleagues did not.

#### 3.3.1 All: Improved management of pain and withdrawal

Across participant groups, the ACS was described as an important resource for improving the management of withdrawal and pain in patients hospitalized with SUD. Specifically, prescribers noted that the ACS helped them feel more comfortable ordering opioids for patients with high tolerance and provided protocols for managing withdrawal. One APP described:

We wind up writing probably more appropriate medication management for this population than previously I was comfortable writing for.

Participant 3

Similarly, a resident physician reported that the ACS assisted in interpretating complex opioid use patterns, guiding safer and more effective treatment:

I’m almost shooting from the hip…trying to figure out what the hell does that mean in terms of how many morphine equivalents you’re getting. And I think the addiction team is much better at doing that. So I feel safer when the replacement plan has come directly from them.

Participant 6

Some nurses also observed positive changes in clinical practice, particularly around the timeliness and appropriateness of treatment for withdrawal. One nurse explained:

[The] addiction medicine consult service really does help. They’ve made a huge impact for many reasons, not only are they just all great people that truly care about their patient population, but I feel like they have a more in-depth understanding than our traditional medicine teams of how to treat withdrawal and how to initiate a proper treatment right away.

Participant 19

Attending physicians echoed this sentiment, noting that the consult team brought both expertise and legitimacy to novel practices which previously had been met with institutional resistance. This attending described a difference before and after the implementation of the ACS, referring to the use of short acting full-agonist opioids to stabilize acute withdrawal from illicit fentanyl:

Because the addiction consult team wasn’t there, and we didn’t have notes from pharmacists saying this was okay to do, we often got pushback from nursing because it was just sort of – to a lot of people, it seemed like this wild approach. So I think having the addiction consult team present and sort of making these recommendations and cosigning this approach has really helped different people throughout the hospital buy into this.

Participant 26

These reflections illustrate how the ACS functions as both a clinical and cultural intervention, equipping providers with the guidance and institutional support to provide evidence-based withdrawal and pain management.

#### 3.3.2 All: Improved discharge planning and care transitions

Interview participants also identified the critical role of the ACS in discharge planning and care transitions for patients with SUD. Many providers described improvement particularly in MOUD initiation and linkage to outpatient treatment and support services. For example, an APP emphasized the ACS role in bridging inpatient and outpatient care:

I really appreciate the ability to link to services as outpatients that we might not have thought of…and just being assured that they have a contact and a way to continue getting Suboxone or hooked into a methadone clinic.

Participant 3

Similarly, an attending physician highlighted how the ACS provides a more reliable framework for safe and coordinated discharge:

I think we’re doing [discharge planning] right a much higher proportion of the time than we used to… there’s a lot more of, like, here’s who you’re going to go to get your Bupe, or the shelter you’re going to go to, or the SNF that’s methadone capable. There’s a lot more connectedness and continuity. If I had to pick one thing that the addiction medicine consult has helped with the most, I think that would be the dispositional planning.

Participant 2

A nurse noted that the inclusion of a dedicated social worker with SUD-specific expertise helped address the complex needs of patients with SUD, which were often unmet by the general floor staff:

It’s helpful that there is a social worker case manager who specifically works with that population, because I think there’s a different array of needs that need to be met. And I think it would be too much for the [floor] case managers and social workers who have so many other challenges that they are trying to help other patients get through… Having dedicated team members who are thinking more holistically… makes me feel less like I’m just saying: “okay, bye, good luck.”

Participant 10

One resident noted the value of the consult team’s role in care transitions, describing how the interdisciplinary team worked together to make the use of hospitalization as a ‘reachable moment’ to engage patients in outpatient support^23^:

The addiction consult service was utilized to essentially get this patient back on suboxone and then set him up with a more consistent outpatient provider, and then the social worker came and helped to find him a more stable housing option that was safe for him to get his suboxone and keep it safe from others. And so it was a way to see kind of how both the physicians and the ancillary service workers on the service, like the social workers and certified recovery specialist, were able to work together to positively impact this patient and take – almost kind of take advantage of the opportunity of him being in the hospital, to get him onto more stable suboxone dose and safely discharge him.

Participant 11

Collectively, these perspectives suggest that the ACS not only improved clinical aspects of discharge, medication reconciliation and outpatient referrals, but also provided a more coordinated, person-centered approach to care. The inclusion of a dedicated SUD-trained social worker was a key structural component enabling these improvements.

#### 3.3.3 All: Reduced moral distress and burnout

Across provider groups, the ACS was reported to alleviate some of the emotional burden of caring for patients with SUD. Participants emphasized that having a specialized team helped distribute the responsibility for complex care while reducing feelings of isolation and helplessness.

An APP described how ACS support reduced emotional strain and modeled compassionate care, while affirming the difficult emotions that often arise when caring for this population:

Sometimes in burnout we lose compassion. And I think the addiction medicine team models compassionate care for patients, meeting them where they are. And I think that’s been really helpful for myself and for my colleagues to see. And it also validates, yeah, [this patient is] really tough to take care of.

Participant 3

A resident similarly reflected on the relief of having clear, expert guidance and shared clinical responsibility, which they perceived also improves patient care:

It helps having a team that specializes in this, and sees this every day, to tell you, okay, this is what we’re gonna do next. And so I think it gives me a lot more support as a resident, and I think it also helps the patient. It helps take some of the burden off of our shoulders, because they’re going in and seeing the patient every day.

Participant 5

Participants also described how the ACS mitigated feelings of futility and moral distress when confronting structural barriers that often fall outside the scope of inpatient care. One nurse explained:

I think some of the moral distress that I feel when caring for patients with SUD, is just this feeling that these problems are so huge and they’re somewhat larger than me, and larger than the patient. They’re just very enormous societal issues and I think we can’t fix these problems in the hospital. But at least it’s not just me bearing witness to this patient suffering alone. And I know also that when I reach out to providers for support, we’re on the same page, and that they’re also clued in to the broader picture… There’s more of an orientation towards compassion and trying to support patients’ wellbeing and less of a judgmental and withholding approach.

Participant 10

An attending physician simply shared:

There’s just something very therapeutic about feeling like this is not your burden to hold alone.

Together, these reflections illustrate how the presence of the ACS not only improves clinical care but also helps clinicians connect with their values of quality, patient-centered care, reducing emotional exhaustion and making the work more sustainable.

#### 3.3.5 Nursing-identified ongoing challenges

Although many nurses expressed appreciation for the ACS and its positive impact on patient care, several described ongoing challenges related to communication, team integration, and barriers to education on SUD. A recurring concern was that nurses were frequently uninformed regarding the ACS’ involvement in patient care. One nurse emphasized the importance of including nursing perspectives, explaining:

More transparency, communication, and including the nurse who was on the ground delivering the care and to hear what the patient is experiencing… We (nurses) are not in the loop about care… We don’t really talk to [the ACS]. They come in, they speak to the patient at times, and then kind of they just leave… We should be doing better with that, like a closed loop communication.

Participant 9

Another nurse echoed this sense of exclusion, noting that while physicians on the care team may be aware of the ACS actions, nursing often is not:

I don’t think the communication is there all the time. Like, maybe the docs know and stuff like that, but I don’t know if the communication is there with the nurses.

Participant 23

This lack of communication often left nurses uncertain about the ACS’ role, scope of work, or even their presence on the unit. This nurse contextualized this as a systemic issue:

In the hospital, as a nurse, we don’t always know what’s going on. We don’t know who’s going into the room… I don’t know who it is… I don’t know what’s happening… I did not have really direct contact or understanding as to what was going on.

Participant 1

In some cases, nurses described cultural attitudes among staff that further complicated the integration of addiction medicine into routine care. One nurse noted the resistance among some long-tenured colleagues, stating:

I do see a lot of nurses who have worked there for a long time who are pretty rude about the addiction medicine consult team, and about patients who have substance use disorders, and just will outwardly talk about these patients as crackheads, or druggies, or addicts. And I think it is more normal for nurses to tell horror stories to each other to blow off steam, but there’s a way to do it where you’re not calling somebody a crackhead. And then, also, sometimes nurses or pharmacists, or whatever, will roll their eyes at some of the things that are being suggested by the addiction medicine consult team, implying that it’s an overstep, and why would they do that for these patients?

Participant 10

The stigmatizing attitudes described may reflect a culture among some clinical staff in which addiction care is de-valued, contributing to less satisfaction or positive engagement with the addiction consult team.

The same nurse elaborated that this disconnect may also be reflected in the structure of inpatient care, where teams often work in silos:

I think if we’re able to have that ongoing communication, that would be really beneficial, in my opinion… because [the ACS] comes in kind of as a separate unit. I have my NP; we’re doing our medical thing and here’s [the ACS] over there. So just everybody being on the same page.

Participant 1

Despite sometimes limited interaction with the team, many of the nurses we interviewed had a sense that the ACS has improved care for patients with SUD but were often not aware of the details of the changes. One nurse shared:

I wish I knew more about what addiction medicine does, but whatever they’re doing, I believe that it’s working.

Participant 23

Nurses expressed desire to be better informed about best practices for managing SUD in the hospital setting, but many described barriers to accessing information or building their knowledge base, particularly amid the demands of bedside work. One nurse explained this in the context of xylazine, a veterinary tranquilizer that is an adulterant in the local street opioid drug supply:

I’m new to the tranq drugs…I’m not familiar with what you would use or not use to make a person comfortable… I probably need a little more education on what’s happening with the tranq, but I don’t have time to sit down and sometimes look it up because I’m running around all the time. So I’m just following orders and doing the best I can…

Participant 1

Another nurse noted that current training opportunities are often optional, limiting their reach:

There’s a lot of opt-in educational opportunities for people who are interested in caring for patients with substance use disorders, but if you’re not opting in …. then you aren’t really getting that education. So I wonder about having more mandatory education, or building it into some of the existing educational requirements for nurses… And I think providing more information around what different medications do, what the goals are when we’re treating withdrawal, some of the other social challenges this population is experiencing.

Participant 10

Others suggested systems-level changes, like incorporating education about SUD care into staff orientation, so that the burden doesn’t fall on individual nurses to seek out resources. One nurse reflected:

I maybe can also take accountability, and say I could take it on myself to learn…Like, what options do we have for unhoused patients? Where can we access that? … But also, just in general, these are things that we should all be able to know…Incorporating that into nursing orientation, how to access resources, so we can better serve our patients holistically too.

Participant 9

Another emphasized the need for a standard of education for nurses around SUD:

There needs to be a level of education around what we’re doing and why.

Participant 19

These accounts illustrate that limited communication, a lack of integration between nursing and the ACS, and gaps in education shaped how nurses experienced the ACS and their ability to engage in SUD-related care.

## 4. DISCUSSION

In this mixed methods study of multidisciplinary perspectives on an inpatient ACS, we found broad agreement across professional groups that the ACS improved key areas of patient care and provider experience, including pain and withdrawal management, discharge planning, and provider moral distress. However, our survey results revealed variability in the experiences of nurses. Interviews contextualized these differences, presenting challenges with communication, team integration, and limited access to education as barriers to nursing engagement with the ACS and SUD care generally. These findings add to the body of literature that ACS improve hospital-based addiction care in multiple domains and also suggest that targeted strategies to support nursing and address team integration are needed to fully realize their potential.^11–13^

To our knowledge, this is one of the first mixed methods assessments of interdisciplinary inpatient teams caring for patients with SUD. While Beckett *et al* previously documented that nurses, relative to physicians and social workers, were less likely to be familiar with an ACS, our study extends these findings by exploring the structural and interpersonal dynamics that shape these differences.^24^ For example, nurses described being excluded from medical team rounds and noted that the while ACS often communicated with the physician and APP teams, their recommendations and clinical decision-making often bypassed nursing colleagues. These communication breakdowns, coupled with limited control over prescriber-led aspects of care, led some nurses to describe feeling disconnected from care decisions. This concern has also been documented in prior work exploring nursing role in addiction care.^25^ These dynamics, while not unique to SUD settings, may be intensified in this context given the complexity of care, stigma, and lack of clear institutional protocol.

One important implication of this work is that ACS implementation efforts must intentionally engage nurses as integral members of the care team. Nurses spend substantial time at the bedside with patients and assess and address symptoms like pain and withdrawal, making them critical in caring for patients with SUD. In our study, nurses generally perceived benefits of the ACS but sought more engagement with the ACS team and clearer insight into clinical decision-making, demonstrating opportunities for improved communication channels embedded in the ACS as well as nurse involvement in ACS implementation and daily functioning. Some nurses also described the ACS as being siloed, which may limit its ability to shift unit norms and standardize care. In addition, our data illustrated variation in nurse perceptions about ACS implementation, with some skepticism or resistance toward the service, especially among those with longer tenure. This suggests that local norms may play a key role in shaping the perception of value of SUD care, and deliberate integration efforts are needed to identify and target sources of hesitancy or resistance (e.g. education deficit, stigma) among nurses. Addressing the gaps will require deliberate implementation strategies to engage nursing in ACS workflows and decision-making. Re-shaping how nurses perceive addiction care can lead to meaningful and lasting improvements in the quality of care received by patients with SUD.

Incorporating nursing staff or a nurse educator into the core ACS team may strengthen multidisciplinary communication and education on best practices for SUD care. Interdisciplinary rounds, case reviews or dedicated ACS liaisons on high-volume units that directly engage bedside nurses may also address these gaps. Models that embed SUD specialists directly into units or offer SUD education to non-specialist providers could further support consistency in care.^26^ Future research should explore how interpersonal and unit-level factors, including stigma and multidisciplinary relationships, can be addressed to optimize ACS implementation.

These interventions are particularly important given that nursing curricula continue to inadequately address SUD, despite a rapidly expanding evidence based for SUD treatment and evolving institutional best practices.^27^ Nursing curricula should adapt to these developments to ensure that new graduates enter practice with the skills to provide evidence-based SUD care. Without institutional investment, nurses may be left underprepared to meaningfully engage with ACS teams and patients with SUD. Additionally, hospitals could consider establishing institutional standards for SUD-related continuing education for nurses or developing specialized programs like the addiction nursing fellowship piloted at Bosom Medical Center, which created a structured pathway to improve nurses’ expertise in SUD care.^28^

Our interviews also emphasize the critical role of the ACS social worker in facilitating successful patient transitions. This reinforces the importance of non-prescribing roles in the ACS and in SUD care more broadly. Although social workers are included on nearly every ACS described in the literature, their contributions are understudied compared to other team members.^10^ In addition to linking patients to post-hospital care and housing, social workers interact extensively with patients during hospitalization, often more than other providers, and, together with peer specialists, play a central role in building trust.^29^ Their unique skills provide forms of support that other clinicians may not be able to offer. Further research examining their impact could help institutions both advocate for the inclusion of social workers in ACS teams, where their role is sometimes undervalued by hospital leadership, and investigate strategies to best support these essential team members.^30^

Many interviewees described the ACS as a source of emotional support and validation in navigating the complex emotional impact of caring for patients with SUD, particularly around pain and withdrawal management and discharge planning. These findings align with prior work that shows ACS can improve patient outcomes in these areas.^11–13^ However, our findings highlight this less explored aspect of ACS impact: its role in supporting provider well-being. Work by Englander *et al* has demonstrated that simply the presence of an ACS may reduce moral distress felt by providers.^31^ In our study, the inability to adequately care for patients with SUD, as well as witnessing stigma towards patients, were described as impacting not only patient care but also staff moral distress. For example, nurse participants relayed wanting to do more for their patients while feeling unequipped to do so. This tension, coupled with reports of difficulty accessing existing (often optional) educational opportunities, suggests potential added value of ACS support beyond improving patient outcomes. This has important implications for workplace sustainability that have not been explored elsewhere. It also provides another avenue for health systems to advocate for ACS funding at their institutions.

Our study has several limitations. Conducted at a single academic hospital in one city, results may not generalize to other settings or policy environments. The 39% survey response rate raises the possibility or nonresponse bias, and self-reported perceptions may be influenced by social desirability. Additionally, interview participants were likely those more engaged in the issue and may not represent the full range of perspectives, particularly those who were less satisfied with ACS. Our qualitative findings may not capture the views of all individuals surveyed. Finally, we did not include patient perspectives, or link perceptions to patient-level outcomes, which may constrain inferences about real world impact.

In conclusion, these findings suggest that while ACS can meaningfully improve care for hospitalized patients with SUD, the perceived impact of this service is uneven across professional groups. Enhancing the impact of ACS requires intentional integration of all frontline providers, especially nurses. Addressing communication barriers, investing in education and role-based support, and promoting multidisciplinary collaboration will be key to expanding the reach and impact of ACS models.

## Figures and Tables

**Table 1:** Survey respondent characteristics

Characteristic	Total (%), n = 311	Attending Physicians (%), n = 49	APP (%), n = 26	Resident Physicians (%), n = 108	Nurses (%), n = 128
**Gender**					
Female	226 (73%)	26 (53%)	22 (85%)	65 (60%)	113 (88%)
Male	81 (26%)	23 (47%)	4 (15%)	40 (37%)	14 (11%)
Other	3 (1%)	0 (0%)	0 (0%)	3 (3%)	1 (1%)
**Race**					
Black	35 (11%)	0 (0%)	2 (8%)	12 (11%)	19 (15%)
White	173 (56%)	28 (57%)	17 (65%)	53 (49%)	75 (59%)
Asian	76 (24%)	17 (35%)	4 (15%)	32 (30%)	23 (18%)
Other	27 (9%)	0 (0%)	0 (0%)	0 (0%)	2 (2%)
**Ethnicity**					
Hispanic/Latinx	16 (5%)	0 (0%)	1 (4%)	8 (7%)	7 (5%)
Non-Hispanic/Latinx	220 (71%)	49 (100%)	25 (96%)	99 (92%)	121 (95%)
**Professional Group**					
Attending Physician	49 (16%)				
APP	26 (8%)				
Nurse	128 (41%)				
Resident Physician	108 (35%)				
**Close Friend or Family with SUD**					
Yes	88 (28%)	5 (10%)	9 (35%)	20 (19%)	54 (42%)
No	204 (66%)	43 (88%)	15 (58%)	78 (72%)	68 (53%)
Did not Answer	19 (6%)	1 (2%)	2 (8%)	10 (9%)	6 (5%)

**Table 2:** Survey results, percent somewhat (4) or strongly agreeing (5), range 1–5

	Total (%), n = 311	Resident (%), n = 108	Attendings (%), n = 49	APP (%), n = 26	Nurses (%), n = 128	p-value (chi-square test)
**Addiction Consult Service support has a positive impact on my ability to provide clinical care**	239 (77%)	96 (89%)	48 (98%)	25 (96%)	69 (54%)	0.000
**Addiction Consult Service support has a positive impact on discharge planning**	246 (79%)	96 (89%)	47 (96%)	23 (88%)	81 (63%)	0.000
**The Addiction Consult Service provides harm reduction education (overdose education, safer use practices, etc.)**	243 (78%)	94 (87%)	47 (96%)	24 (92%)	77 (60%)	0.000
**Addiction Consult Service support positively impacts care for people who continue to use drugs**	249 (80%)	96 (89%)	47 (96%)	26 (100%)	81 (63%)	0.000
**My communications with the Addiction Consult Service are helpful**	221 (71%)	95 (88%)	45 (92%)	25 (96%)	55 (43%)	0.000
**Our hospital administration values addiction care**	208 (67%)	68 (63%)	39 (80%)	23 (88%)	79 (62%)	.167

**Table 3:** Qualitative interview participant characteristics

Characteristic	Total, n = 20 (%)
**Gender**	
Female	12 (60%)
Male	7 (35%)
Non-binary	1 (5%)
**Race**	
White	13 (65%)
Black	5 (25%)
Asian	2 (10%)
**Ethnicity**	
H/L	0 (0%)
Not H/L	20 (100%)
**Years of experience**	
<1 year	5 (25%)
1–5 years	12 (60%)
6–10 years	2 (10%)
10 + years	1 (5%)
**Professional Group**	
Nurse	7 (35%)
Attending Physician	4 (20%)
Resident Physician	4 (20%)
APP	2 (10%)
Social worker	2 (10%)
Peer	1 (5%)

## Data Availability

Data not available / The data that has been used is confidential. Due to the sensitive nature of the questions asked in this study, participants were assured raw data would remain confidential and would not be shared.
